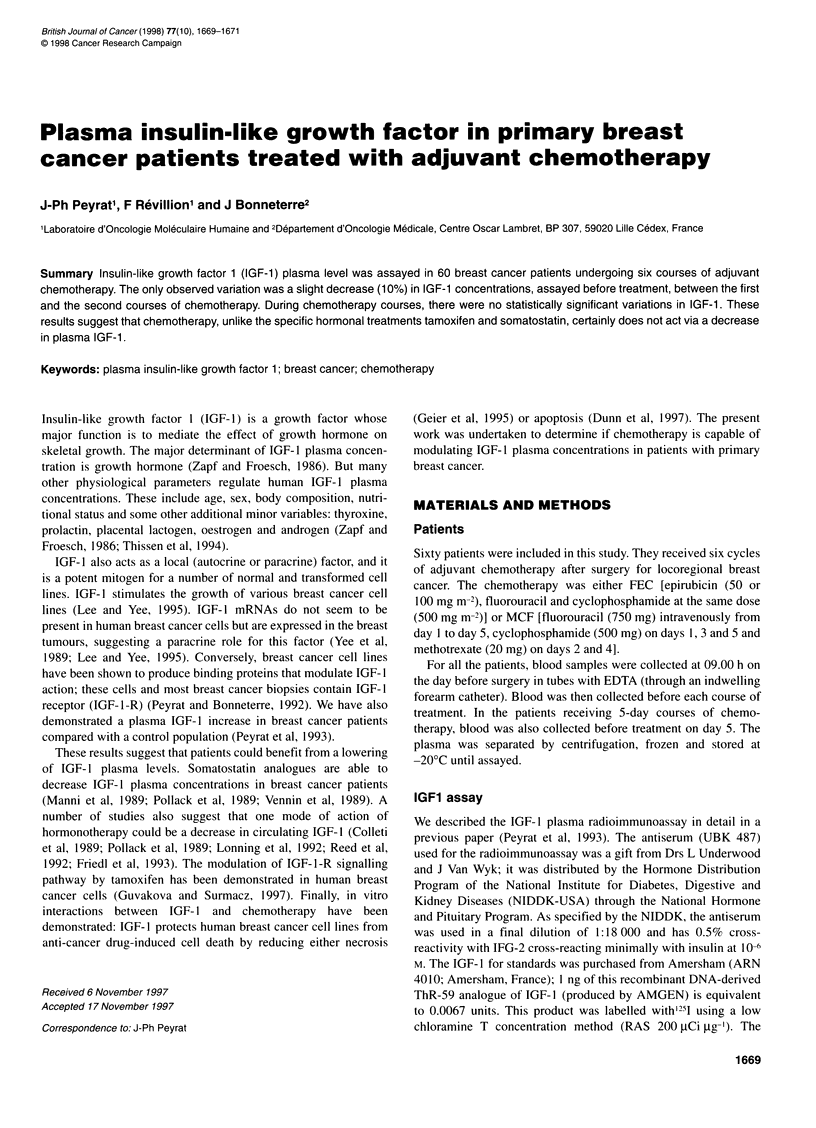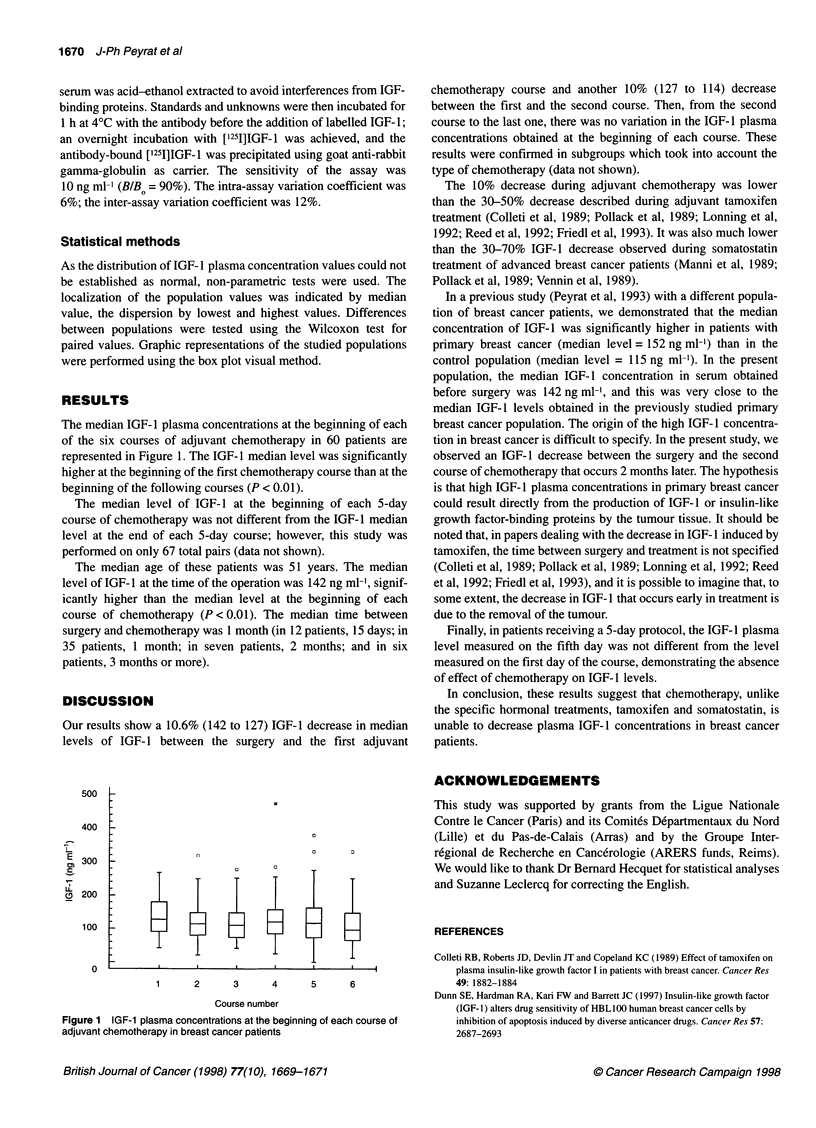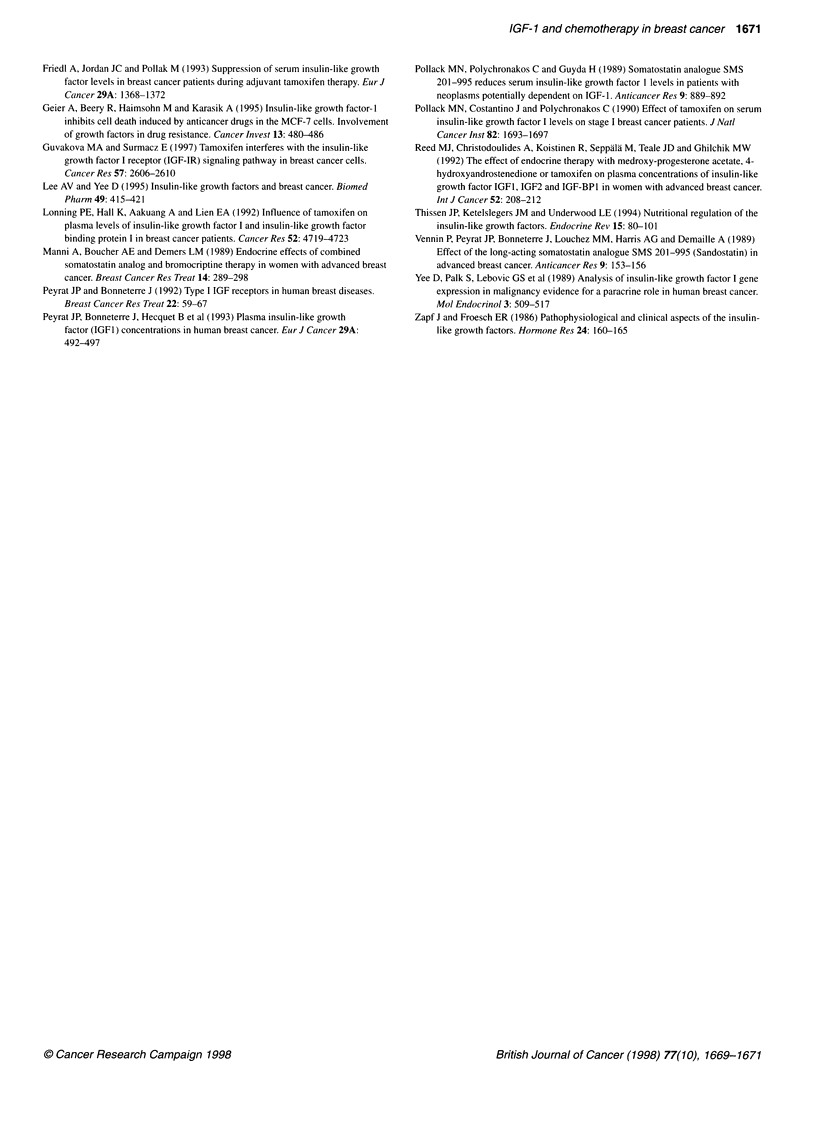# Plasma insulin-like growth factor in primary breast cancer patients treated with adjuvant chemotherapy.

**DOI:** 10.1038/bjc.1998.274

**Published:** 1998-05

**Authors:** J. P. Peyrat, F. RÃ©villion, J. Bonneterre

**Affiliations:** Laboratoire d'Oncologie MolÃ©culaire Humaine, Centre Oscar Lambret, Lille, France.

## Abstract

Insulin-like growth factor 1 (IGF-1) plasma level was assayed in 60 breast cancer patients undergoing six courses of adjuvant chemotherapy. The only observed variation was a slight decrease (10%) in IGF-1 concentrations, assayed before treatment, between the first and the second courses of chemotherapy. During chemotherapy courses, there were no statistically significant variations in IGF-1. These results suggest that chemotherapy, unlike the specific hormonal treatments tamoxifen and somatostatin, certainly does not act via a decrease in plasma IGF-1.


British Journal of Cancer (1998) 77(10), 1669–1671
© 1998 Cancer Research Campaign

### Plasma insulin-like growth factor in primary breast cancer patients treated with adjuvant chemotherapy

**J-Ph Peyrat[1], F Révillion[1] and J Bonneterre[2]**

[1]Laboratoire d'Oncologie Moléculaire Humaine and [2]Département d'Oncologie Médicale, Centre Oscar Lambret, BP 307, 59020 Lille Cédex, France

**Summary** Insulin-like growth factor 1 (IGF-1) plasma level was assayed in 60 breast cancer patients undergoing six courses of adjuvant chemotherapy. The only observed variation was a slight decrease (10%) in IGF-1 concentrations, assayed before treatment, between the first and the second courses of chemotherapy. During chemotherapy courses, there were no statistically significant variations in IGF-1. These results suggest that chemotherapy, unlike the specific hormonal treatments tamoxifen and somatostatin, certainly does not act via a decrease in plasma IGF-1.

**Keywords:** plasma insulin-like growth factor 1; breast cancer; chemotherapy

Insulin-like growth factor 1 (IGF-1) is a growth factor whose major function is to mediate the effect of growth hormone on skeletal growth. The major determinant of IGF-1 plasma concentration is growth hormone (Zapf and Froesch, 1986). But many other physiological parameters regulate human IGF-1 plasma concentrations. These include age, sex, body composition, nutritional status and some other additional minor variables: thyroxine, prolactin, placental lactogen, oestrogen and androgen (Zapf and Froesch, 1986; Thissen et al, 1994).

IGF-1 also acts as a local (autocrine or paracrine) factor, and it is a potent mitogen for a number of normal and transformed cell lines. IGF-1 stimulates the growth of various breast cancer cell lines (Lee and Yee, 1995). IGF-1 mRNAs do not seem to be present in human breast cancer cells but are expressed in the breast tumours, suggesting a paracrine role for this factor (Yee et al, 1989; Lee and Yee, 1995). Conversely, breast cancer cell lines have been shown to produce binding proteins that modulate IGF-1 action; these cells and most breast cancer biopsies contain IGF-1 receptor (IGF-1-R) (Peyrat and Bonneterre, 1992). We have also demonstrated a plasma IGF-1 increase in breast cancer patients compared with a control population (Peyrat et al, 1993).

These results suggest that patients could benefit from a lowering of IGF-1 plasma levels. Somatostatin analogues are able to decrease IGF-1 plasma concentrations in breast cancer patients (Manni et al, 1989; Pollack et al, 1989; Vennin et al, 1989). A number of studies also suggest that one mode of action of hormonotherapy could be a decrease in circulating IGF-1 (Colleti et al, 1989; Pollack et al, 1989; Lonning et al, 1992; Reed et al, 1992; Friedl et al, 1993). The modulation of IGF-1-R signalling pathway by tamoxifen has been demonstrated in human breast cancer cells (Guvakova and Surmacz, 1997). Finally, in vitro interactions between IGF-1 and chemotherapy have been demonstrated: IGF-1 protects human breast cancer cell lines from anti-cancer drug-induced cell death by reducing either necrosis (Geier et al, 1995) or apoptosis (Dunn et al, 1997). The present work was undertaken to determine if chemotherapy is capable of modulating IGF-1 plasma concentrations in patients with primary breast cancer.

*Received 6 November 1997*
*Accepted 17 November 1997*

*Correspondence to:* J-Ph Peyrat

### MATERIALS AND METHODS

### Patients

Sixty patients were included in this study. They received six cycles of adjuvant chemotherapy after surgery for locoregional breast cancer. The chemotherapy was either FEC [epirubicin (50 or 100 mg $m^{-2}$), fluorouracil and cyclophosphamide at the same dose (500 mg $m^{-2}$)] or MCF [fluorouracil (750 mg) intravenously from day 1 to day 5, cyclophosphamide (500 mg) on days 1, 3 and 5 and methotrexate (20 mg) on days 2 and 4].

For all the patients, blood samples were collected at 09.00 h on the day before surgery in tubes with EDTA (through an indwelling forearm catheter). Blood was then collected before each course of treatment. In the patients receiving 5-day courses of chemotherapy, blood was also collected before treatment on day 5. The plasma was separated by centrifugation, frozen and stored at –20°C until assayed.

### IGF1 assay

We described the IGF-1 plasma radioimmunoassay in detail in a previous paper (Peyrat et al, 1993). The antiserum (UBK 487) used for the radioimmunoassay was a gift from Drs L Underwood and J Van Wyk; it was distributed by the Hormone Distribution Program of the National Institute for Diabetes, Digestive and Kidney Diseases (NIDDK-USA) through the National Hormone and Pituitary Program. As specified by the NIDDK, the antiserum was used in a final dilution of 1:18 000 and has 0.5% cross-reactivity with IFG-2 cross-reacting minimally with insulin at $10^{-6}$ M. The IGF-1 for standards was purchased from Amersham (ARN 4010; Amersham, France); 1 ng of this recombinant DNA-derived ThR-59 analogue of IGF-1 (produced by AMGEN) is equivalent to 0.0067 units. This product was labelled with $^{125}I$ using a low chloramine T concentration method (RAS 200 μCi $\mu g^{-1}$). The serum was acid–ethanol extracted to avoid interferences from IGF-binding proteins. Standards and unknowns were then incubated for 1 h at 4°C with the antibody before the addition of labelled IGF-1; an overnight incubation with [$^{125}$I]IGF-1 was achieved, and the antibody-bound [$^{125}$I]IGF-1 was precipitated using goat anti-rabbit gamma-globulin as carrier. The sensitivity of the assay was 10 ng ml$^{-1}$ ($B/B_0$ = 90%). The intra-assay variation coefficient was 6%; the inter-assay variation coefficient was 12%.

### Statistical methods

As the distribution of IGF-1 plasma concentration values could not be established as normal, non-parametric tests were used. The localization of the population values was indicated by median value, the dispersion by lowest and highest values. Differences between populations were tested using the Wilcoxon test for paired values. Graphic representations of the studied populations were performed using the box plot visual method.

### RESULTS

The median IGF-1 plasma concentrations at the beginning of each of the six courses of adjuvant chemotherapy in 60 patients are represented in Figure 1. The IGF-1 median level was significantly higher at the beginning of the first chemotherapy course than at the beginning of the following courses ($P < 0.01$).

The median level of IGF-1 at the beginning of each 5-day course of chemotherapy was not different from the IGF-1 median level at the end of each 5-day course; however, this study was performed on only 67 total pairs (data not shown).

The median age of these patients was 51 years. The median level of IGF-1 at the time of the operation was 142 ng ml$^{-1}$, significantly higher than the median level at the beginning of each course of chemotherapy ($P < 0.01$). The median time between surgery and chemotherapy was 1 month (in 12 patients, 15 days; in 35 patients, 1 month; in seven patients, 2 months; and in six patients, 3 months or more).

### DISCUSSION

Our results show a 10.6% (142 to 127) IGF-1 decrease in median levels of IGF-1 between the surgery and the first adjuvant chemotherapy course and another 10% (127 to 114) decrease between the first and the second course. Then, from the second course to the last one, there was no variation in the IGF-1 plasma concentrations obtained at the beginning of each course. These results were confirmed in subgroups which took into account the type of chemotherapy (data not shown).

The 10% decrease during adjuvant chemotherapy was lower than the 30–50% decrease described during adjuvant tamoxifen treatment (Colleti et al, 1989; Pollack et al, 1989; Lonning et al, 1992; Reed et al, 1992; Friedl et al, 1993). It was also much lower than the 30–70% IGF-1 decrease observed during somatostatin treatment of advanced breast cancer patients (Manni et al, 1989; Pollack et al, 1989; Vennin et al, 1989).

In a previous study (Peyrat et al, 1993) with a different population of breast cancer patients, we demonstrated that the median concentration of IGF-1 was significantly higher in patients with primary breast cancer (median level = 152 ng ml$^{-1}$) than in the control population (median level = 115 ng ml$^{-1}$). In the present population, the median IGF-1 concentration in serum obtained before surgery was 142 ng ml$^{-1}$, and this was very close to the median IGF-1 levels obtained in the previously studied primary breast cancer population. The origin of the high IGF-1 concentration in breast cancer is difficult to specify. In the present study, we observed an IGF-1 decrease between the surgery and the second course of chemotherapy that occurs 2 months later. The hypothesis is that high IGF-1 plasma concentrations in primary breast cancer could result directly from the production of IGF-1 or insulin-like growth factor-binding proteins by the tumour tissue. It should be noted that, in papers dealing with the decrease in IGF-1 induced by tamoxifen, the time between surgery and treatment is not specified (Colleti et al, 1989; Pollack et al, 1989; Lonning et al, 1992; Reed et al, 1992; Friedl et al, 1993), and it is possible to imagine that, to some extent, the decrease in IGF-1 that occurs early in treatment is due to the removal of the tumour.

Finally, in patients receiving a 5-day protocol, the IGF-1 plasma level measured on the fifth day was not different from the level measured on the first day of the course, demonstrating the absence of effect of chemotherapy on IGF-1 levels.

In conclusion, these results suggest that chemotherapy, unlike the specific hormonal treatments, tamoxifen and somatostatin, is unable to decrease plasma IGF-1 concentrations in breast cancer patients.

**Figure 1** IGF-1 plasma concentrations at the beginning of each course of adjuvant chemotherapy in breast cancer patients

### ACKNOWLEDGEMENTS

This study was supported by grants from the Ligue Nationale Contre le Cancer (Paris) and its Comités Départmentaux du Nord (Lille) et du Pas-de-Calais (Arras) and by the Groupe Inter-régional de Recherche en Cancérologie (ARERS funds, Reims). We would like to thank Dr Bernard Hecquet for statistical analyses and Suzanne Leclercq for correcting the English.

### REFERENCES

Colleti RB, Roberts JD, Devlin JT and Copeland KC (1989) Effect of tamoxifen on plasma insulin-like growth factor I in patients with breast cancer. *Cancer Res* **49**: 1882–1884

Dunn SE, Hardman RA, Kari FW and Barrett JC (1997) Insulin-like growth factor (IGF-1) alters drug sensitivity of HBL100 human breast cancer cells by inhibition of apoptosis induced by diverse anticancer drugs. *Cancer Res* **57**: 2687–2693

© Cancer Research Campaign 1998

Friedl A, Jordan JC and Pollak M (1993) Suppression of serum insulin-like growth factor levels in breast cancer patients during adjuvant tamoxifen therapy. *Eur J Cancer* **29A**: 1368–1372

Geier A, Beery R, Haimsohn M and Karasik A (1995) Insulin-like growth factor-1 inhibits cell death induced by anticancer drugs in the MCF-7 cells. Involvement of growth factors in drug resistance. *Cancer Invest* **13**: 480–486

Guvakova MA and Surmacz E (1997) Tamoxifen interferes with the insulin-like growth factor I receptor (IGF-IR) signaling pathway in breast cancer cells. *Cancer Res* **57**: 2606–2610

Lee AV and Yee D (1995) Insulin-like growth factors and breast cancer. *Biomed Pharm* **49**: 415–421

Lonning PE, Hall K, Aakuang A and Lien EA (1992) Influence of tamoxifen on plasma levels of insulin-like growth factor I and insulin-like growth factor binding protein I in breast cancer patients. *Cancer Res* **52**: 4719–4723

Manni A, Boucher AE and Demers LM (1989) Endocrine effects of combined somatostatin analog and bromocriptine therapy in women with advanced breast cancer. *Breast Cancer Res Treat* **14**: 289–298

Peyrat JP and Bonneterre J (1992) Type I IGF receptors in human breast diseases. *Breast Cancer Res Treat* **22**: 59–67

Peyrat JP, Bonneterre J, Hecquet B et al (1993) Plasma insulin-like growth factor (IGF1) concentrations in human breast cancer. *Eur J Cancer* **29A**: 492–497

Pollack MN, Polychronakos C and Guyda H (1989) Somatostatin analogue SMS 201–995 reduces serum insulin-like growth factor 1 levels in patients with neoplasms potentially dependent on IGF-1. *Anticancer Res* **9**: 889–892

Pollack MN, Costantino J and Polychronakos C (1990) Effect of tamoxifen on serum insulin-like growth factor I levels on stage I breast cancer patients. *J Natl Cancer Inst* **82**: 1693–1697

Reed MJ, Christodoulides A, Koistinen R, Seppälä M, Teale JD and Ghilchik MW (1992) The effect of endocrine therapy with medroxy-progesterone acetate, 4-hydroxyandrostenedione or tamoxifen on plasma concentrations of insulin-like growth factor IGF1, IGF2 and IGF-BP1 in women with advanced breast cancer. *Int J Cancer* **52**: 208–212

Thissen JP, Ketelslegers JM and Underwood LE (1994) Nutritional regulation of the insulin-like growth factors. *Endocrine Rev* **15**: 80–101

Vennin P, Peyrat JP, Bonneterre J, Louchez MM, Harris AG and Demaille A (1989) Effect of the long-acting somatostatin analogue SMS 201–995 (Sandostatin) in advanced breast cancer. *Anticancer Res* **9**: 153–156

Yee D, Palk S, Lebovic GS et al (1989) Analysis of insulin-like growth factor I gene expression in malignancy evidence for a paracrine role in human breast cancer. *Mol Endocrinol* **3**: 509–517

Zapf J and Froesch ER (1986) Pathophysiological and clinical aspects of the insulin-like growth factors. *Hormone Res* **24**: 160–165

© Cancer Research Campaign 1998